# Determinants of Vessel Targeting in Vasculitis

**DOI:** 10.1080/17402520400001652

**Published:** 2004

**Authors:** Gary S. Hoffman

**Affiliations:** Harold C. Schott Chair of Rheumatic and Immunologic Diseases Director, Center for Vasculitis Care and Research Cleveland OH USA

## Abstract

Studies of autoimmune diseases have not yet elucidated why certain organs or vessels become the objects of injury while others are spared. This paper will explore the hypothesis that important differences exist in regions of the aorta that determine vulnerability to diseases, such as atherosclerosis, aortitis, giant cell arteritis and Takayasu's disease. The reader is invited to reassess; (1) whether the aorta is indeed a single homogeneous structure, and (2) whether the initial stage of aortitis (and indeed other diseases considered “autoimmune”) may be primarily due to acquired alterations of substrate, that influence unique immune profiles, which by themselves may not be pathogenic. Disease susceptibility and patterns are influenced by many factors that are inborn and acquired. Examples include genetic background, gender, ethnicity, aging, prior and concomitant illnesses, habits, diet, toxin and environmental exposures. Studies of vascular diseases must assess how such variables may affect regional differences in endothelial cells, subendothelial matrix, vascular smooth muscle and the response of each to a variety of stimuli.


*Clinical & Developmental Immunology*, September/December 2004, Vol. 11 (3/4), pp. 275–279

Taylor & Francis healthsciences

### Determinants of Vessel Targeting in Vasculitis*

GARY S. HOFFMAN[†]

*Harold C. Schott Chair of Rheumatic and Immunologic Diseases, Director, Center for Vasculitis Care and Research, Cleveland Clinic Foundation A50, Cleveland, OH 44195, USA*

Studies of autoimmune diseases have not yet elucidated why certain organs or vessels become the objects of injury while others are spared. This paper will explore the hypothesis that important differences exist in regions of the aorta that determine vulnerability to diseases, such as atherosclerosis, aortitis, giant cell arteritis and Takayasu's disease. The reader is invited to reassess; (1) whether the aorta is indeed a single homogeneous structure, and (2) whether the initial stage of aortitis (and indeed other diseases considered "autoimmune") may be primarily due to acquired alterations of substrate, that influence unique immune profiles, which by themselves may not be pathogenic. Disease susceptibility and patterns are influenced by many factors that are inborn and acquired. Examples include genetic background, gender, ethnicity, aging, prior and concomitant illnesses, habits, diet, toxin and environmental exposures. Studies of vascular diseases must assess how such variables may affect regional differences in endothelial cells, subendothelial matrix, vascular smooth muscle and the response of each to a variety of stimuli.

### GIANT CELL, TAKAYASU'S ARTERITIS AND IDIOPATHIC AORTITIS

Giant cell (GCA) and Takayasu's arteritis (TA) are granulomatous inflammatory diseases of large arteries that share a similar, and some would argue, the same profile of targets in regard to vessel size and distribution. Both may be complicated by inflammatory aneurysms that are predominantly thoracic (Hoffman, 2003). However, in one illness (GCA) the mean age at presentation is $>70$ years, while in the other (TA) it is about 25 years (Kerr *et al.*, 1994). In patients with idiopathic aortitis (IA), which may or may not have granulomatous histopathology, age is less polarized and there is often absence of systemic illness (Rojo-Leyva *et al.*, 2000). Such patients may only come to attention because of the hemodynamic effects of aortic regurgitation and root dilatation. Aortitis may be recognized only after specimens are reviewed from aortic aneurysm surgeries (Fig. 1). We have reported the characteristics of patients with IA and compared them to patients with atherosclerosis, cystic medial necrosis, GCA and TA. Of 48 inflammatory aneurysms in our IA cohort, 96% were thoracic. In contrast, among 798 *non-inflammatory aortic aneurysms*, 69% were in the abdominal region and were due principally to atherosclerosis (i.e. inflammation only around atheromatous plaque, not diffuse). This striking trend for inflammatory aneurysms to be located in the thoracic region has been confirmed in surveys of large vessel pathology from post-mortem examinations. Aortitis of uncertain etiology is recognized in up to 8% of all post-mortems. When authors have restricted analyses to aortitis with aneurysms, inflammatory aneurysms were almost always noted in the thoracic region (Ostberg, 1973; Bickerstaff *et al.*, 1982; Svensjo *et al.*, 1996).

The most common cause of thoracic aortic aneurysms is not aortitis; it is cystic medionecrosis (Pomerance *et al.*, 1997). Cystic medionecrosis, in the absence of atherosclerosis, is far more common in the thoracic than the abdominal aorta, where it generally co-exists with and may result, in part, from atherosclerotic injury. Patchy atherosclerosis may be common in post-mortem thoracic aorta specimens in the elderly, however, atherosclerosis alone is an uncommon cause of thoracic aneurysm formation.

These observations emphasize variations in vulnerability to different diseases, within the same vessel, which we will go on to see is *not homogenous* throughout its course.

*Supported in part by the HCS Foundation.
[†]Corresponding author. Tel.: +1 (216) 445 6996. Fax: +1 (216) 445 7569.

ISSN 1740-2522 print/ISSN 1740-2530 online © 2004 Taylor & Francis Ltd
DOI: 10.1080/17402520400001652

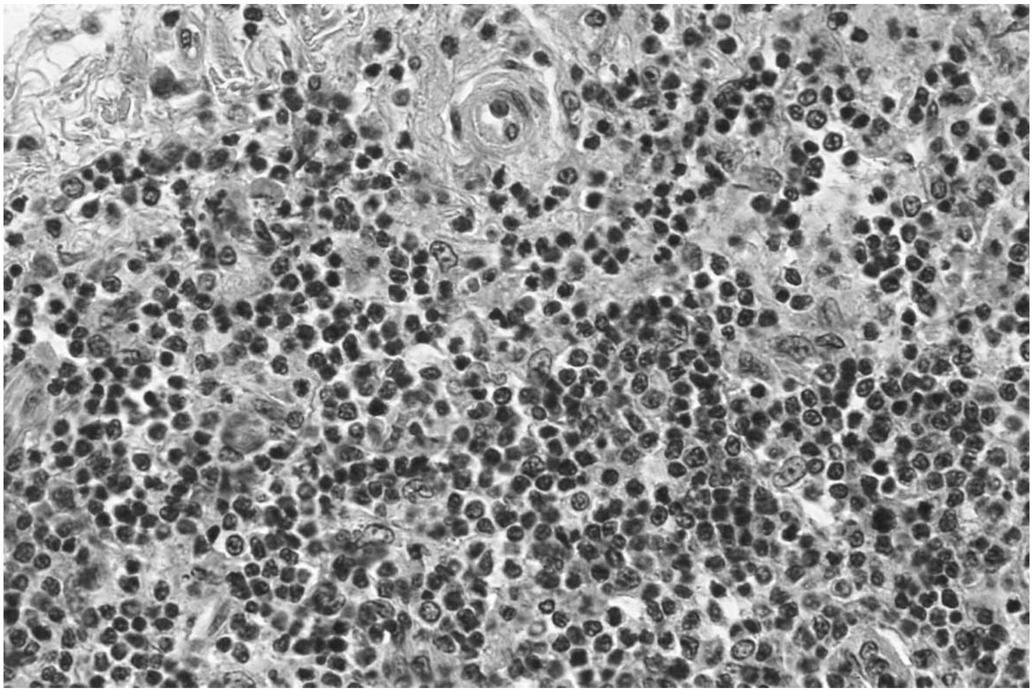

FIGURE 1 Aortitis was discovered after surgery in a 67-year-old man who did not have symptoms of a systemic inflammatory illness. Surgical intervention was required for aortic root dilatation and consequent severe aortic regurgitation (300 × mag).

### HOW IS THE AORTA DIFFERENT THROUGHOUT ITS REGIONS?

The foregoing discussion implies that this single tubular structure, the aorta, must have inherent variations in its content and/or responsiveness to injury to account for regional differences in disease patterns. In addition, apart from Takayasu's arteritis, inflammatory aortic aneurysms are found in older adults. Does age provide opportunities for change within regions of the aorta that make it more vulnerable to inflammatory injury? Are there elements of immunological senescence that may respond to altered substrate to produce aortitis? What are the implications for younger patients with aortitis? Although answers to these questions are not readily available, regional differences within the aorta are well known and will be considered (Table I).

TABLE I Relative differences between the thoracic and abdominal aorta

| Feature | Thoracic region | Abdominal region |
|---|---|---|
| Wall thickness | Greater | |
| Wall diameter | 30–40% greater | |
| Elastic fiber concentration | Greater (decreases distal to root) | |
| Derivation of SMCs* | Neural crest ectoderm | Mesoderm |
| Smooth muscle cells-media$^{inner}$ | | Greater concentration |
| Vasa vasorum (media) | Numerous | Few to absent |
| Disease predilections | Inflammatory cystic medial necrosis | Diffuse atherosclerosis |

*Smooth muscle cells.

### Anatomic differences

The thoracic aorta is wider and has a thicker wall diameter than that of more distal regions. In man, the aortic arch wall is about 1.5 mm thick, whereas the abdominal aortic wall is ≤1 mm thick (Okuyama *et al.*, 1988).

### Vessel wall perfusion, the vasa vasorum

The concentration of aortic vasa vasorum is about 9.4 vessels/mm$^2$ in the thick-walled thoracic region and only 1.9 vessels/mm$^2$ in the abdominal region (Fig. 2).

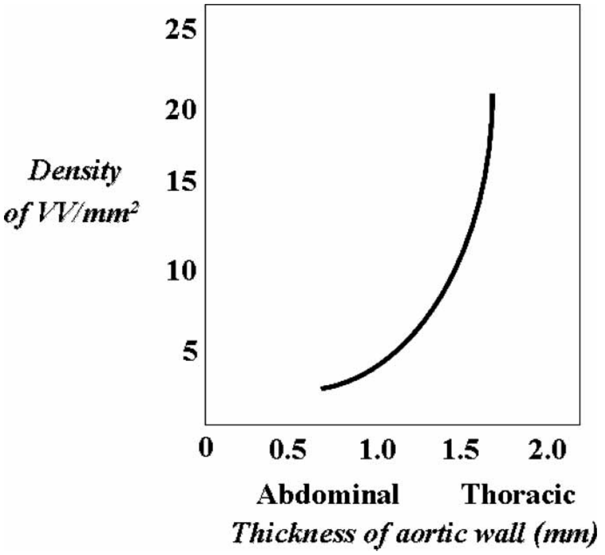

FIGURE 2 In concert with decreased thickness of the aortic wall, vasa vasorum (vv) become less dense from proximal to distal aorta (from Okuyama *et al.*, 1988).

As wall thickness increases with disease, especially in the example of atherosclerosis, there is concordant penetration of the vasa vasorum into the vascular media and even into the intima (Schutte, 1968; Okuyama *et al.*, 1988).

### Aorta collagen and elastin

The synthesis of collagen and elastin (Fisher *et al.*, 1980), as well as thickness of elastic membranes and number of elastic laminae (Sokolis *et al.*, 2002), diminish with increased distance from the aortic root. Elastin, the main extracellular matrix component in arteries, is synthesized by vascular smooth muscle cells and plays both a structural and developmental role in arterial morphogenesis. Animals that lack the gene for elastin have abnormal vascular smooth muscle cell (SMC) proliferation and neointimal thickening. Elastin is therefore a regulatory protein, influencing proliferation and migration of its cell of origin (SMC) (Karnik *et al.*, 2003). With aging, the aorta develops increasing degrees of elastin fragmentation and degeneration, concurrent with increased collagen content. The functional consequences are well understood. The result is loss of elasticity and stiffening (Schlatmann and Becker, 1977). Whether changes in elastin influence immunogenicity is less certain.

### Animal model of atherosclerosis

In a study reported in 1966, Haimovici and Maier (Haimovici and Maier, 1966) addressed the issue of aortic heterogeneity in a study of dogs given a diet intended to induce atherosclerosis, a process that is most obvious in the abdominal aorta. Prior to providing the atherogenic diet, the authors switched segments of thoracic and abdominal aorta between these inbred dogs. The abdominal-located thoracic aorta did not develop atherosclerosis and the thoracic-located abdominal aorta still developed atherosclerosis. Thus, apart from location and properties of flow, inherent differences in aortic wall anatomy and physiology determined disease vulnerability.

### Embryologic differences

Studies of aortic embryogenesis in animals reveal that neural crest cells contribute to the formation of smooth muscle cells (SMCs) in the aortic trunk, proximal arch, pulmonary artery trunk, but not in the more distal parts of the aorta. In contrast, abdominal aortic SMCs are primarily derived from mesoderm. These basic differences imply that unique genetic programs may be responsible for variable responses of SMCs to cytokine stimulation in the thoracic and abdominal aorta (Gadson *et al.*, 1997).

### Understanding differences at a genetic level

Absi *et al.* recently utilized gene expression techniques to more fully explore this issue. They asked why aortic aneurysms in the thoracic region are usually due to cystic medial necrosis, while atherosclerosis is the primary cause of abdominal aortic aneurysms? Striking differences in gene expression patterns were identified between areas of unaffected thoracic and abdominal aorta, as well as between segments of aneurysms from both regions (Absi *et al.*, 2003).

### Role of exogenous pathogens

The possible role of undiscovered infection in GCA, TA and IA is an area of great controversy. Nonetheless, in other settings infection is a known cause of aortitis and demonstrates site selectivity. Aortitis may result from *Staphylococcus aureus*, *Salmonella*, fungi, tuberculous or other organisms (Oz *et al.*, 1989). The patterns of idiopathic aortitis and infectious aortitis are often similar. For example, syphilitic aortitis involves the thoracic aorta in ~75% of cases (ascending 46%, transverse arch 25%), while infrequently affecting the aorta distal to the renal arteries (Heggtveit, 1964).

Pathogens possess adhesion molecules and may produce unique products with selective tissue affinities. Selective adhesion may account for differences in targeting end-organs (Patti *et al.*, 1994). The fact that products of pathogens may act at sites remote from their origin allows for injury to occur in the absence of the pathogen itself. Alternatively, pathogens may be effectively cleared, but have induced a response to self-Ag (molecular mimicry) or caused alterations of self-Ag, that lead it to be perceived as foreign (neoantigen). It is also possible that if autoimmunity were triggered by an infectious agent, the critical factor leading to illness may not be one's ability to clear the agent, but the inability to down-regulate the immune response.

Variations in immune response are likely to explain the broad range of outcomes observed following a variety infections. Hepatitis viruses, parvovirus B19 and Epstein-Barr virus (EBV) are well known for the diversity of illness phenotypes. For example, in children, parvovirus B19 may cause erythema infectiosum, but in other individuals produce red-cell aplasia, aplastic anemia, a Still's disease-like illness, a rheumatoid arthritis-like illness, or hydrops fetalis during pregnancy. The virus appears to be the same, but the end result is extraordinarily different.

### IMMUNE FUNCTION CHANGES WITH AGE

While it is likely that acquired changes in the vessel wall play a role in determining patterns of vasculitis, it is also likely that acquired changes in immune function may be required for disease expression. Certain vasculitides are clearly age and gender biased. Age and gender factors include changes in hormonal function. Eighty per cent of patients with Kawasaki disease are less than 5 years old, and boys are affected 1.5 times as often as girls. TA affects mostly women of reproductive age and GCA is seen in individuals whose mean age is 74 years, with women being affected about twice as often as men. Immune senescence may be especially relevant to GCA. Increasing age is associated with decline in thymic function, impairment of T cell activation, defective

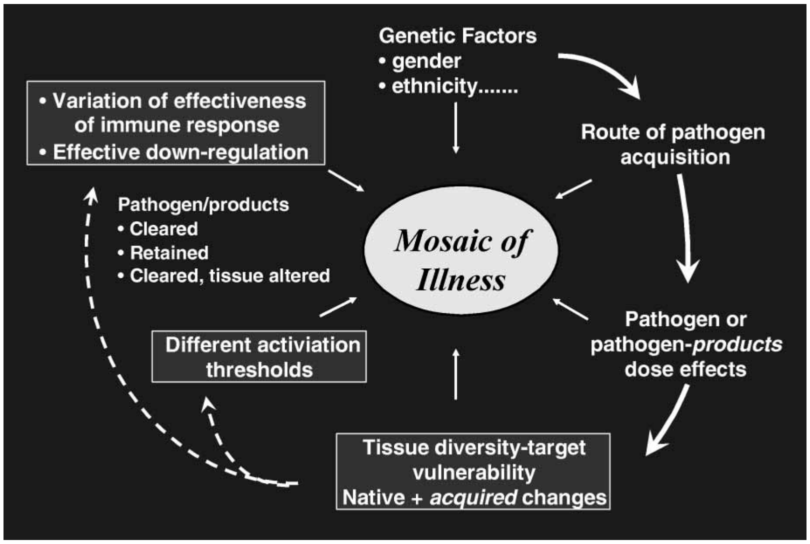

FIGURE 3 Conceptual mosaic for organ targeting. Pathogenic factors in immune-mediated diseases may be endogenous and/or exogenous. Whether disease results, may in part be related to general or tissue-specific host susceptibility factors. Determinants of organ targeting may reside within the target itself, and include unique native or acquired characteristics (e.g. ligands for pathogens, age-related changes, post-infectious changes etc), that influence a pathogen's (organisms or molecules) adhesion, retention, and penetration. In health, pathogenic factors would be cleared, the immune response would be appropriately down-regulated and normal structure and function would be restored (reprinted from Hoffman, 2003; with permission of Wiley and Sons).

generation of T cell memory, decline in specific antibody responses, defects in apoptosis and defects in antigen presentation and processing (Weyand and Goronzy, 1997).

### CONCLUSIONS

Certain basic pathogenic components of "vasculitis" require greater study. There is a need to acquire more complete knowledge of the sites that are the object of injury, before injury actually occurs. While it is important to study the immune reactive/inflammatory cells and their products within the affected site, it is unlikely that that approach alone will reveal the etiology of these disorders. Studies of the "target" might include distinguishing molecular and genetic differences between controls and patients in regards to elements of tissue structure, antigens and response to injury. It is likely that we will find differences in the form of mosaic patterns, and not single genes or molecules within the affected sites (Fig. 3).

If the initial events in the pathogenesis of certain vasculitides are related to changes in the vessel wall and not due primarily to aberrations in immune function, microarray and proteomic techniques may provide unique insights into susceptibility factors imparted by age, ethnicity and gender. For example, GCA is frequent among people whose origins are Northern European or Icelandic. However, it is relatively uncommon in African-Americans and Asians. Important differences in the "targeted" vessels in these groups may enhance or determine disease susceptibility. Microarray and proteomic techniques are also currently being applied to study pattern variation within temporal arteries in GCA and normal controls. These tools are providing opportunities to better understand the role of the targeted tissue in vasculitis.

### *References*

Absi, T.S., Sundt, T.M., III, Tung, W.S., Moon, M., *et al.* (2003) "Altered patterns of gene expression distinguishing ascending aortic aneurysms from abdominal aortic aneurysms: complementary DNA expression profiling in the molecular characterization of aortic disease", *J. Thorac. Cardiovasc. Surg.* **126**, 344–357.

Bickerstaff, L.K., Pairolero, P.C., Hollier, L.H., Melton, J., *et al.* (1982) "Thoracic aortic aneurysms: A population based study", *Surg* **92**, 1103–1108.

Fisher, G.M., Swain, M.L. and Cherian, K. (1980) "Increased collagen and elastin synthesis in experimental atherosclerosis in the rabbit. Variation in synthesis among major vessels", *Atherosclerosis* **35**, 11–20.

Gadson, P.F., Dalton, M.L., Patterson, E., Svoboda, D.D., *et al.* (1997) "Differential response of mesoderm- and neural crest-derived smooth muscle to TGF-beta1: regulation of c-myb and alpha1 procollagen genes", *Exp. Cell Res.* **230**, 169–180.

Haimovici, H. and Maier, N. (1966) "Role of arterial tissue susceptibility in experimental canine atherosclerosis", *J. Atherosclerosis Res.* **6**, 62–74.

Heggtveit, H.A. (1964) "Syphilitic aortitis. A clinicopathologic autopsy study of 100 cases, 1950–1960", *Circulation* **29**, 346–355.

Hoffman, G.S. (2003) "Large vessel vasculitis: unresolved issues", *Arthritis Rheum.* **48**, 2406–2414.

Karnik, S.K., Brooke, B.S., Bayes-Genes, A., Sorensen, L., Wythe, J.D., Schwartz, R.S., Keating, M.T. and Li, D.Y. (2003) "A critical role for elastin signaling in vascular morphogenesis and disease", *Development* **130**, 411–423.

Kerr, G.S., Hallahan, C.W., Giordano, J., Leavitt, R.Y., Fauci, A.S., Rottem, M. and Hoffman, G.S. (1994) "Takayasu arteritis", *Ann. Intern. Med.* **120**, 919–929.

Okuyama, K., Yaginuma, G., Takahashi, T., Sasaki, H. and Mori, S. (1988) "The development of vasa vasorum of the human aorta in various conditions. A morphometric study", *Arch. Pathol. Lab. Med.* **112**, 721–725.

Ostberg, G. (1973) "An arteritis with special reference to polymyalgia rheumatica", *ACTA Pathol. Microbiol. Scand.* **237**(Suppl.), 1–59.

Oz, M.C., Brener, B.J., Buda, J.A., Todd, G., Brenner, R.W., *et al.* (1989) "A ten year experience with bacterial aortitis", *J. Vasc. Surg.* **10**, 439–449.

Patti, J.M., Allen, B.L., McGavin, M.J. and Hook, M. (1994) "MSCRAMM-mediated adherence of microrganisms to host tissues", *Annu. Rev. Microbiol.* 585–617.

Pomerance, A., Yacoub, M.H. and Gula, G. (1997) "The surgical pathology of thoracic aortic aneurysms", *Histopathology* **1**, 257–276.

Rojo-Leyva, F., Ratliff, N.B., Cosgrove, D.M., III and Hoffman, G.S. (2000) “Study of 52 patients with idiopathic aortitis from a cohort of 1,204 surgical cases”, *Arthritis Rheum.* **43**, 901–907.

Schlatmann, T. and Becker, A. (1977) “Pathogenesis of dissecting aneurysm of the aorta. Comparartive histopathologic study of significance of medial changes”, *Am. J. Cardiol.* **39**, 21–26.

Schutte, H.E. (1968) “Changes in the vasa vasorum of the atherosclerotic aortic wall”, *Angiologica* **5**, 210–222.

Sokolis, D.P., Boudoulas, H., Kavantzas, N.G., Kostomitsopulous, N., Agapitos, E.V. and Karayannacos, P.E. (2002) “A morphometric study of the structural characteristics of the aorta in pigs using an image analysis method”, *Anat. Histol. Embryol. Vet. Med. Series C* **31**, 21–30.

Svensjo, S., Bengtsson, H. and Bergqvist, D. (1996) “Thoracic and thoracoabdominal aortic aneurysm and dissection: Investigation based on autopsy”, *Br. J. Surg.* **83**, 68–71.

Weyand, C.M. and Goronzy, J.J. (1997) “Multisystem interactions in the pathogenesis of vasculitis”, *Curr. Opin. Rheumatol.* **9**, 3–11.

